# Motor imagery ability in children and adolescents with cerebral palsy: a systematic review and evidence map

**DOI:** 10.3389/fneur.2024.1325548

**Published:** 2024-02-06

**Authors:** José Fierro-Marrero, Alejandro Corujo-Merino, Roy La Touche, Sergio Lerma-Lara

**Affiliations:** ^1^Departamento de Fisioterapia, Centro Superior de Estudios Universitarios La Salle, Universidad Autónoma de Madrid, Aravaca, Madrid, Spain; ^2^Motion in Brains Research Group, Centro Superior de Estudios Universitarios La Salle, Universidad Autónoma de Madrid, Aravaca, Madrid, Spain; ^3^Instituto de Dolor Craneofacial y Neuromusculoesquelético (INDCRAN), Madrid, Spain

**Keywords:** cerebral palsy, motor imagery, mental chronometry, laterality judgment, body schema

## Abstract

**Background:**

Cerebral palsy (CP) refers to a group of permanent movement and posture disorders. Motor imagery (MI) therapy is known to provide potential benefits, but data on MI ability in children and adolescents with CP is lacking.

**Objective:**

A systematic review was performed to explore MI abilities in children and adolescents with CP compared to typically developed (TD) subjects.

**Methods:**

We searched on PubMed, Web of Science (WOS), EBSCO, Google Scholar, and PEDro including observational studies. Methodological quality was assessed with the modified Newcastle–Ottawa Scale and evidence map was created to synthesize the evidence qualitatively and quantitatively.

**Results:**

Seven cross-sectional studies were selected, which included 174 patients with CP and 321 TD subjects. Three studies explored explicit MI, two MI–execution synchrony, and four implicit MI domains. Methodological quality ranged from 6 to 8 stars. Moderate evidence supported the absence of differences in vividness between the groups. As there was only limited evidence, establishing a clear direction for the results was not possible, especially for the capacity to generate MI, mental chronometry features, and MI–execution synchrony domains. Moderate evidence supported a lower efficiency in cases for hand recognition, derived from a lower accuracy rate, while reaction time remained similar between the two groups. Moderate evidence indicated that patients with CP and TD controls showed similar features on whole-body recognition.

**Conclusion:**

Moderate evidence suggests that patients with CP present a reduced ability in hand recognition, which is not observed for whole-body recognition compared to healthy controls. Severe limitations concerning sample size calculations and validity of assessment tools clearly limits establishing a direction of results, especially for explicit MI and MI-Execution synchrony domains. Further research is needed to address these limitations to enhance our comprehension of MI abilities in children, which is crucial for prescribing suitable MI-based therapies in this child population.

## 1 Introduction

Cerebral palsy (CP) is a group of permanent developmental disorders of movement and posture, causing a limitation of activity (1).

There are several definitions of CP proposed in the literature, and they are all based on the presence of permanent motor and posture disorders, which are usually accompanied by other cognitive, sensorial, and behavioural disorders, or even with epilepsy (2). These impairments are attributed to non-progressive disorders that occur in the developing fetal or infant brain (2). However, the aetiology of CP, which is critical for its diagnosis, shows that prenatal influences appear to play a more significant role in its manifestation, while perinatal factors contribute to a lesser extent (3).

Cerebral palsy has a global prevalence of 2.11 per 1,000 live births (4), exhibiting an increasing trend from 1988 to 2019 (5). The economic costs of CP can reach billions of dollars per patient over a lifetime (6).

Cerebral palsy causes significant functional limitations, as 61.8% of the patients are found to exhibit difficulties conforming to those between level II and IV of the Gross Motor Function Classification System (GMFCS) and 43.7% have no independent gait (7). Children and adolescents with CP exhibit increased medio-lateral deviations in gait compared with typically developed (TD) individuals, thus presenting with an increased gait performance difficulty (8). Patients with CP not only experience deficits in the execution of movement, but also in movement planning (9). Optimal movement planning facilitates the achievement of efficient movement execution and appears to improve with age (10).

The mental representation of movement is a complex process that involves the preparation, planning, and organization of movement (11). This process occurs unconsciously but can be intentionally elicited through various techniques, such as motor imagery (MI), action observation (AO), or visual feedback. MI is a cerebral process of constructing a motor action without the actual execution (12). This process is developed through the involvement of perceptual-sensory mechanisms that facilitate the formulation of motor actions, which involve the working memory (13). MI processes may occur implicitly or explicitly. Although implicit and explicit MI are conceptually, theoretically, and practically distinct, they are found to recruit similar sensorimotor neural networks associated with movement (14).

Implicit MI is concerned with motor representations that occur in prospective action judgements and in perceptual decisions regarding behaviour (15). Implicit MI tasks require participants to make judgements by making use of visual stimuli that automatically (and implicitly) activate the mental simulation of actions (16). An example of implicit MI is when an individual performs a mental transformation of their own hand to solve a task in an attempt to find congruence with the presented hand (17). Laterality judgement tasks are the most commonly used for assessing implicit MI ability (18).

Implicit MI is typically evaluated in terms of accuracy and reaction time with images of different body parts (19). Hand laterality judgement (HLJ) task, a widely employed task, requires the participants to identify whether the presented hand image is the left or right hand. Although controversial evidence exists suggesting that certain individuals can complete these body recognition tasks without employing MI strategies (20, 21), it would be useful to explore this ability in children and adolescents with CP, as body recognition tasks can also serve as a therapeutic tool for rehabilitation (22).

Explicit MI involves consciously mentally performing an action (23). MI outcome measures have been covered extensively in a recent systematic review (19). These measures include the capacity to generate MI, its vividness, and mental chronometry (time required to imagine) (24–26). Explicit MI, usually evaluated in terms of mental chronometry, can also be contrasted with execution performance, to identify the synchrony between both the abilities (MI–execution synchrony) (19).

Considering that MI abilities develop between the ages of 5 and 12 years (27), it would be of interest to analyse this capacity in children and adolescents with CP to determine who would benefit most from MI interventions. Existing evidence supports employing MI and AO therapies to enhance functional abilities in adults with neurological and musculoskeletal disorders (28). Unfortunately, limited evidence exists on the efficacy of MI therapy for CP patients, with only one reported randomized controlled trial (29). Notably, MI therapy did not significantly enhance functional performance compared to conventional physiotherapy in these patients. This stresses the need for further research to clarify the effectiveness of MI as a therapeutic tool. Evaluating MI abilities in children and adolescents with CP may help clinicians to determine the potential benefits of MI-based therapy for improving functional abilities in patients with CP. Indirect evidence from healthy adults suggests that a higher capacity to generate MI correlates with marked functional enhancements after MI interventions (30). These findings should also be contrasted in CP patients.

After conducting a preliminary search across several databases, we found no systematic reviews summarizing MI abilities in children and adolescents with CP. To address this knowledge gap, we conducted a systematic review with the aim of evaluating explicit MI, MI-execution synchrony, and implicit MI in children and adolescents with CP in comparison to TD subjects.

## 2 Methods

This systematic review was registered in the International Prospective Register of Systematic Reviews (PROSPERO) under the registration number CRD42022345725. It was conducted following the Preferred Reporting Items for Systematic Reviews and Meta-analysis guidelines recommended by Moher et al. (31).

### 2.1 Selection criteria

The selection criteria for study inclusion were based on population of interest, control of interest, outcome measures, and study design.

#### 2.1.1 Cases

The case subjects selected for the studies were children (6–12 years) and/or adolescents (13–18 years) who had been diagnosed with CP. As mentioned earlier, the aetiology of CP appears to be more strongly influenced by prenatal factors than by perinatal features. While stroke, traumatic brain injuries, and other events are considered perinatal factors in CP development, their exclusive presence is not a definitive indicator of the presence of CP. Confirming a CP diagnosis requires the identification of additional signs and symptoms (2). Therefore, this review will focus solely on cases with a confirmed diagnosis of CP.

#### 2.1.2 Controls

Cases should be compared with a control group of TD children (6–12 years) and/or adolescents (13–18 years).

#### 2.1.3 Outcome measures

The outcome measures of interest included: (1) capacity to generate MI; (2) vividness during MI; and (3) mental chronometry. These outcome measures are categorized as explicit MI capacities and were extracted regardless of MI perspective (first/third person) or modality (kinaesthetic, visual internal, or visual external). MI–execution synchrony outcome measures were also included as an outcome, including the performance over- or underestimation coefficients (ratio or difference between MI and execution time), and the variance analysis of MI and execution time data. The following outcome measures categorized as implicit MI capacities were also included: (4) hand recognition through HLJ task; (5) feet recognition through a foot laterality judgement task; and (6) whole-body recognition tasks. Eligible data could be presented either in terms of accuracy, reaction time, or efficiency indexes.

#### 2.1.4 Study design

Observational studies were eligible for inclusion.

### 2.2 Data sources and searches

Systematic searches were performed in PubMed, Web of Science (WOS), EBSCO, Google Scholar, and PEDro databases on 7 July 2022. Additional records were identified through manual searches until 28 September 2022. We conducted an updated systematic search in PubMed on 13 December 2023 and identified additional records through manual searches until the same date.

Non-scientific articles, study protocols, and articles without full text were excluded. No restrictions were applied on language. The screening process was performed manually, analyzing the title, abstract, and full text.

Search engines, databases, equations, and registries retrieved are presented in the Supplementary material.

### 2.3 Data extraction

The following information was extracted from the included studies: author(s), publication date, study design, groups examined, sample size, bilateral cerebral palsy (BCP) or unilateral cerebral palsy (UCP), children and/or adolescents, age, and other demographic features. Only outcome measures of interest were extracted and categorized into MI assessment domain, task, and outcome measure. The results were narratively summarized and the performance between cases and controls were noted.

Neuroimaging data were not included in the extraction process. The extracted information was presented in both narrative and tabular formats.

### 2.4 Methodological quality assessment

The methodological quality of cross-sectional studies was evaluated using the Newcastle–Ottawa Scale (NOS), adapted to cross-sectional studies (32). This scale presents a moderate inter-rater reliability (33). The scale consists of seven items divided into three dimensions (selection, comparability, and outcome), with scores ranging from 0 to 10 stars.

Two independent reviewers assessed the methodological quality of all the included studies using the same methodology. The level of agreement was analysed using Cohen’s Kappa coefficient. Agreement scores were categorized as almost perfect if κ coefficients were in the range 0.81–1.00; substantial if 0.61–0.80; moderate if 0.41–0.6; fair if 0.21–0.4; slight if 0.00–0.20; and < 0.00 as poor (34). Disagreements between reviewers were resolved by consensus and by including a third reviewer.

A categorization of methodological quality was established for the included studies following the procedure employed by Elizagaray-García et al. as follows (35):

1. *Good quality*: 3 or 4 stars in selection, 1 or 2 stars in comparability, and 2 or 3 stars in outcomes.
2. *Moderate quality*: 2 stars in selection, 1 or 2 stars in comparability, and 2 or 3 stars in outcomes.
3. *Poor quality*: 0 or 1 star in selection, 0 stars in comparability, and 0 or 1 star in outcomes.

### 2.5 Synthesis of evidence

The synthesis of evidence was based on an adaptation method proposed by La Touche et al. (36) from the system developed by Van Tulder et al. (37). The levels of evidence were categorized as follows:

*“No evidence”*: Absence of observational studies, including cross-sectional or longitudinal studies.

*“Contradictory evidence”*: Inconsistent findings among multiple studies (cross-sectional and longitudinal observational studies).

*“Limited evidence”*: One low-quality case–control study and/or cohort study and/or at least two cross-sectional studies of low quality. For the present study, an additional modification was made – including the presence of one or two low-quality and/or one or two moderate-quality cross-sectional studies.

*“Moderate evidence”*: Consistent findings from multiple low-quality case–control studies and/or cohort studies and/or cross-sectional studies or one high-quality case–control study and/or cohort study. An additional modification was applied in this category for the present study: including the presence of one or two high-quality cross-sectional studies.

*“Strong evidence”*: Consistent findings among multiple high-quality case–control studies and/or cohort studies and/or cross-sectional studies (at least three of these studies).

### 2.6 Qualitative evidence mapping

A qualitative evidence map was developed to visually summarize the obtained results. The following parameters were employed to develop the evidence map:

*X*-axis: This axis was divided into three categories based on the methodological quality assessment method employed by Elizagaray-García et al. (35). Studies were categorized on this axis according to their respective methodological quality ratings.

*Y*-axis: This axis represented the outcome measures and measurement tools employed in the reviewed studies. Studies were positioned along this axis based on the outcome measures they assessed.

Figure size: The size of each bubble corresponded to the number of children and adolescents with CP analyzed in each study.

Bubble color: Each bubble was assigned a colour indicating the results of the comparison between patients with CP and TD subjects. Three colours were employed to represent different types of information reported in the studies: (1) patients with CP presented better performance in blue; (2) no performance difference between groups in yellow; and (3) patients with CP presented poorer performance in red.

Bubble external pattern: Each bubble included an external pattern indicating the population of CP included in the study. Three categories were used: (1) unilateral cerebral palsy (UCP) with a vertical line; (2) bilateral cerebral palsy (BCP) with a horizontal line; and (3) UCP and BCP with a cross pattern.

### 2.7 Quantitative evidence mapping

Available quantitative data from the included studies were extracted and presented as a forest plot in order to graphically represent the direction of the different MI abilities between CP and TD subjects. This graphical representation would aid in observing, the direction tendency of the MI abilities, along with qualitative synthesis and evidence map.

Available data was extracted from text, tables, and graphics (using WebPlotDigitizer online software^1^). Transformations were performed if needed for transforming the data into mean and SD. Standardized mean differences, with the Hedges’ *g* (38), were calculated and displayed in a forest plot.

All these procedures were conducted in R Studio software version 2023.06.0 + 421, employing the R version 4.3.1 (39). Calculations for Hedges’ g was performed with the package “metafor” 3.8.2 version (40).

## 3 Results

### 3.1 Selection process

**Figure 1:**
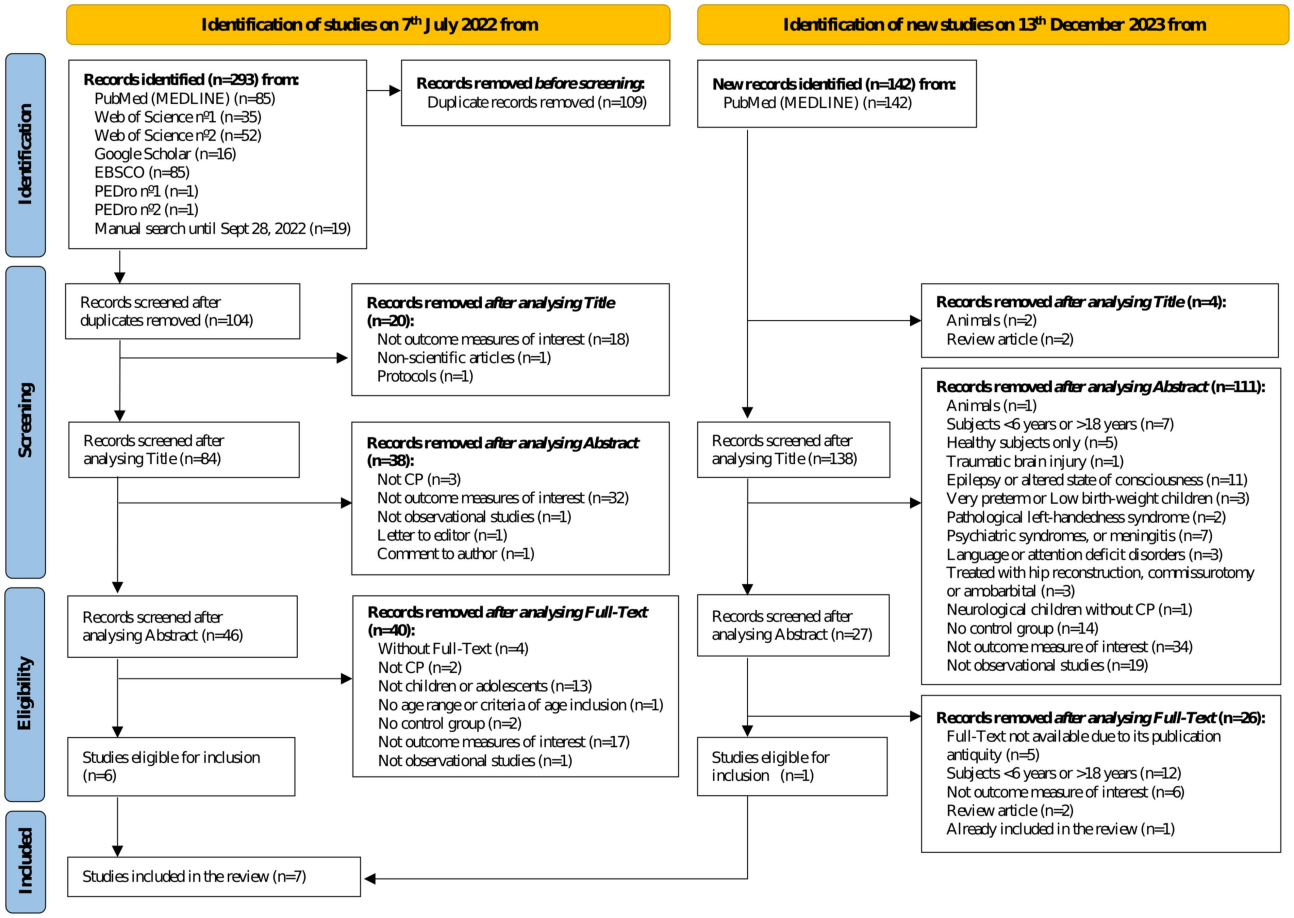
Flow chart of selection process according to PRISMA.

The process of identification, screening, and inclusion of studies is shown in Figure 1.

### 3.2 Data extraction

#### 3.2.1 Population characteristics

A total of seven cross-sectional studies were included, accounting for a total of 174 children and adolescents with CP and 321 TD individuals (29, 41–46). Four of these studies included only patients with UCP, having 120 cases (29, 41–43). One study included only patients with BCP, with a total of 30 cases (44). Two studies included both UCP and BCP patients, accounting for 24 cases (45, 46).

#### 3.2.2 Outcome measures assessed

##### 3.2.2.1 Explicit MI

Explicit MI was assessed in terms of capacity to generate MI from kinaesthetic, visual internal, and visual external modalities (29) using the Movement Imagery Questionnaire for Children. Vividness was also evaluated from kinaesthetic and visual external modalities, employing the Vividness of Movement Imagery Questionnaire Revised 2nd version (45). Mental chronometry was also analysed from unilateral UL tasks (42).

##### 3.2.2.2 MI–execution synchrony

MI–execution synchrony was explored in terms of performance overestimation on the basis of Delta coefficient for LL tasks (29). Delta values >0 indicate that participants employ less time to imagine than executing, suggesting that they overestimate their real performance. Values <0 suggest that participants underestimate their performance, as they would require greater times to imagine than for executing the task.


$$\text{Delta time}=\left[\frac{\text{Execution}\;\text{time}-MI\;\text{time}}{\left(\text{Execution}\;\text{time}+MI\;\text{time}\right)/2\;}\right]$$

Additionally, one study explored the variance distribution of MI and execution chronometry across CP and TD subjects, for unilateral UL tasks (42).

##### 3.2.2.3 Implicit MI

Implicit MI was determined for hand recognition with the HLJ task (41, 43, 44, 46). The studies evaluated this ability in terms of accuracy (41, 43, 46), reaction time (41, 43), and efficiency (44). Whole-body recognition was also assessed in terms of efficiency (44) (Table 1).

**Table 1:** Characteristics of included studies.

Study and *design*	Population	MI assessment domain		Task	Outcome measure	Results	
						Summary	Performance direction
Butti et al., 2019 *Cross-sectional*	Case group (n = 30): BCP; children and adolescents; 7–18 years; FSIQ 41–115. Control group (*n* = 30) TD; children and adolescents; 7–18 years	Implicit MI	Hand recognition	HLJ task – Palm-back viewpoints for both hands, no rotations applied.	Efficiency (IE-Index^‡^)	Cases performed than controls.	CP < TD
			Whole-body recognition task	Recognition of same-different images of whole-body recognition task	Efficiency (IE-Index^‡^)	No differences appeared between groups	CP ≈ TD
Di Vita et al., 2020 *Cross-sectional*	Case group (*n* = 12): UCP and BCP; children; 8–11 years Control group (*n* = 65); TD; children; 8–11 years	Implicit MI	Hand recognition	HLJ task – Back viewpoints for both hands, at 0°, 45°, 90°, 270°, 315° of rotation.	Accuracy (n° correct responses)	Cases presented less accuracy than controls.	CP < TD
Errante et al., 2019 *Cross-sectional*	Case group (*n* = 10): UCP; children and adolescents; 9–14 years; WPPSI ≥70 Control group (*n* = 12): TD; children and adolescents; 9–14 years	Explicit MI	Temporal features	Reaching, grasping, and placing an object, with the *more-affected hand*	Mental chronometry (s)	No differences appeared between groups	CP ≈ TD
				Reaching, grasping, and placing an object, with the *less-affected hand*	Mental chronometry (s)	No differences appeared between groups	CP ≈ TD
		MI-Execution synchrony	Variance distribution of MI and Executed temporal features	Reaching, grasping, and placing an object, with the *more-affected hand*	Mental and Executed chronometry (s)	No differences appeared between groups	CP ≈ TD
				Reaching, grasping, and placing an object, with the *less-affected hand*	Mental and Executed chronometry (s)	No differences appeared between groups	CP ≈ TD
Galli et al., 2022 *Cross-sectional*	Case group (*n* = 12): UCP (*n* = 5) and BCP (*n* = 7); children; 7–12 years; FIQ 79–133. Control group (*n* = 12): TD; children; 7–12 years	Explicit MI	Vividness of MI from kinaesthetic modality	VMIQ-2	1–5 points	No differences appeared between groups	CP ≈ TD
			Vividness of MI from visual external modality	VMIQ-2	1–5 points	No differences appeared between groups	CP ≈ TD
Gözaçan Karabulut et al., 2022 *Cross-sectional*	Case group 1 (*n* = 17): UCP; children and adolescents; 7–18 years; MMSE-C 28–37 Case group 2 (*n* = 17): UCP; children and adolescents; 7–18 years; MMSE-C 28–37 Control group (*n* = 17): TD; children and adolescents; 7–18 years	Explicit MI	Capacity to generate MI from kinaesthetic modality	MIQ-C	1–7 points	Cases presented poorer capacity than controls	CP < TD
			Capacity to generate MI from visual internal modality	MIQ-C	1–7 points	Cases presented poorer capacity than controls	CP < TD
			Capacity to generate MI from visual external modality	MIQ-C	1–7 points	Cases presented poorer capacity than controls	CP < TD
		MI-Execution synchrony	Performance overestimation	TUG	Delta coefficient*	Cases greatly overestimated their performance compared to controls	CP < TD
				10MWT	Delta coefficient*	Cases greatly overestimated their performance compared to controls	CP < TD
Souto et al., 2020 *Cross-sectional*	Case group (*n* = 57): Right UCP (*n* = 32) and left UCP (*n* = 25); children and adolescents; 6–14 years. No cut-off values stablished for IQ measures. Control group (*n* = 175): TD; children and adolescents; 6–14 years	Implicit MI	Hand recognition	HLJ task - Palm-back viewpoints for both hands, with 90° rotation increase	Accuracy	Both case groups presented poorer accuracy than controls	CP < TD
					Reaction time (ms)	No differences appeared between groups	CP ≈ TD
Williams et al., 2021 *Cross-sectional*	Case group (*n* = 19): Left UCP (*n* = 12), right UCP (*n* = 7); children and adolescents; 7–13 years. WASI 76–107 Control group (*n* = 10): TD; children; 8–12 years	Implicit MI	Hand recognition	HLJ task – Palm-back viewpoints for both hands, with 45° rotation increase	Accuracy (%)	No differences were observed between groups	CP ≈ TD
					Reaction time (ms)	No differences were observed between groups	CP ≈ TD

10MWT, 10-Meter Walk Test; BCP, bilateral cerebral palsy; CP, cerebral palsy; FIQ, full intelligence quotient; FSIQ, full-scale intelligent quotient; HLJ, hand laterality judgment; IE-Index, inverse efficiency index; MI, motor imagery; MMSE-C, mini-mental state examination for children; MIQ-C, movement imagery questionnaire for children; TD, typically developed; TUG, timed-up and go test; UCP, unilateral cerebral palsy; VMIQ-2, vividness of movement imagery questionnaire revised second version; WASI, Wechsler abbreviated scale of intelligence; WPPSI, Wechsler preschool and primary scale of intelligence. ^‡^Inverse efficiency index: $IE\;\text{Index}=\left[\frac{\text{Reaction Time}\;\left(ms\right)}{\text{Accuracy}\;\left(\%\text{Correct responses}\right)}\right]$. *Delta coefficient was analyzed with chronometry data (s): $\text{Delta time}=\left[\frac{\text{Execution}\;\text{time}-MI\;\text{time}}{\left(\text{Execution}\;\text{time}+MI\;\text{time}\right)/2\;}\right]$.

Efficiency was measured with the inverse efficiency (IE) index:


$$IE\;\text{Index}=\left[\frac{\text{Reaction Time}\;\left(ms\right)}{\text{Accuracy}\;\left(\%\;\text{Correct responses}\right)\;}\right]$$

### 3.3 Methodological quality assessment

Among the seven cross-sectional studies, four presented good methodological quality (41, 44–46), two moderate methodological quality (29, 43), and one poor methodological quality (42). They accounted for an overall methodological quality of 7.29 ± 0.76 (6–8 stars). An almost perfect level of inter-rater agreement was observed on the NOS scale adapted for cross-sectional studies (κ = 0.832). The results of the methodological quality analysis are shown in Table 2.

**Table 2:** Quality assessment of cross-sectional studies with Newcastle-Ottawa Scale.

Study	Selection item n°1	Selection item n°2	Selection item n°3	Selection item n°4	Comparability	Outcome item n°1	Outcome item n°2	Total	Methodological quality
Butti et al, 2019	★	_	★	★★	★★	★	★	8/10	Good
Di Vita et al., 2020	★	_	★	★★	★★	★	★	8/10	Good
Errante et al, 2019	★	_	★	★★	★★	_	★	7/10	Poor
Galli et al, 2022	★	_	★	★★	★★	★	★	8/10	Good
Karabulut et al, 2022	★	_	★	_	★★	★	★	6/10	Moderate
Souto et al, 2020	★	_	★	★	★★	★	★	7/10	Good
Williams et al, 2021	_	_	★	★	★★	★★	★	7/10	Moderate

★ represents 1 point, ★★ represents 2 points.

### 3.4 Evidence synthesis

#### 3.4.1 Explicit MI – capacity to generate MI

“*Limited evidence*” from one moderate-quality study (29) shows that patients with CP exhibited poorer capacity to generate MI from kinaesthetic, visual internal, and visual external modalities compared to controls.

#### 3.4.2 Explicit MI – vividness

*“Moderate evidence”* from one good-quality study (45), indicates that patients with CP and TD present similar MI vividness when kinaesthetic and visual external modalities were evaluated.

#### 3.4.3 Explicit MI – mental chronometry

*“Limited evidence”* from one poor-quality study (42) suggests that patients with CP and TD subjects exhibit similar mental chronometry features during unilateral UL tasks, with either the more or less affected UL.

#### 3.4.4 MI-execution synchrony – performance overestimation

*“Limited evidence”* from one moderate-quality study (29) shows that patients with CP greatly overestimate their performance compared to TD subjects in LL tasks (timed-up and go test, and 10 meter walk test).

#### 3.4.5 MI-execution synchrony – variance distribution of MI and execution chronometry

*“Limited evidence”* from one poor-quality study suggests that patients with CP and TD subjects took similar times for MI and execution of unilateral UL tasks, with either the more or less affected UL (42).

#### 3.4.6 Implicit MI – hand recognition: accuracy

*“Moderate evidence”* from two good-quality studies (41, 46) demonstrates that patients with CP presented poorer accuracy than TD controls.

*“Limited evidence”* from one moderate-quality study (43) indicates that patients with CP and TD controls exhibited similar accuracy.

#### 3.4.7 Implicit MI – hand recognition – reaction time

*“Moderate evidence”* from one good-quality study (41) supports that patients with CP and TD controls had similar reaction times.

*“Limited evidence”* from one moderate-quality study (43) shows that patients with CP and TD controls exhibited similar reaction times.

#### 3.4.8 Implicit MI – hand recognition – efficiency

*“Moderate evidence”* from one good-quality (44) study found a poorer efficiency in patients with CP compared to TD subjects.

#### 3.4.9 Implicit MI - whole-body recognition – efficiency

*“Moderate evidence”* from one good-quality (44) identified similar efficiency values between patients with CP and TD subjects.

### 3.5 Qualitative evidence mapping.

**Figure 2:**
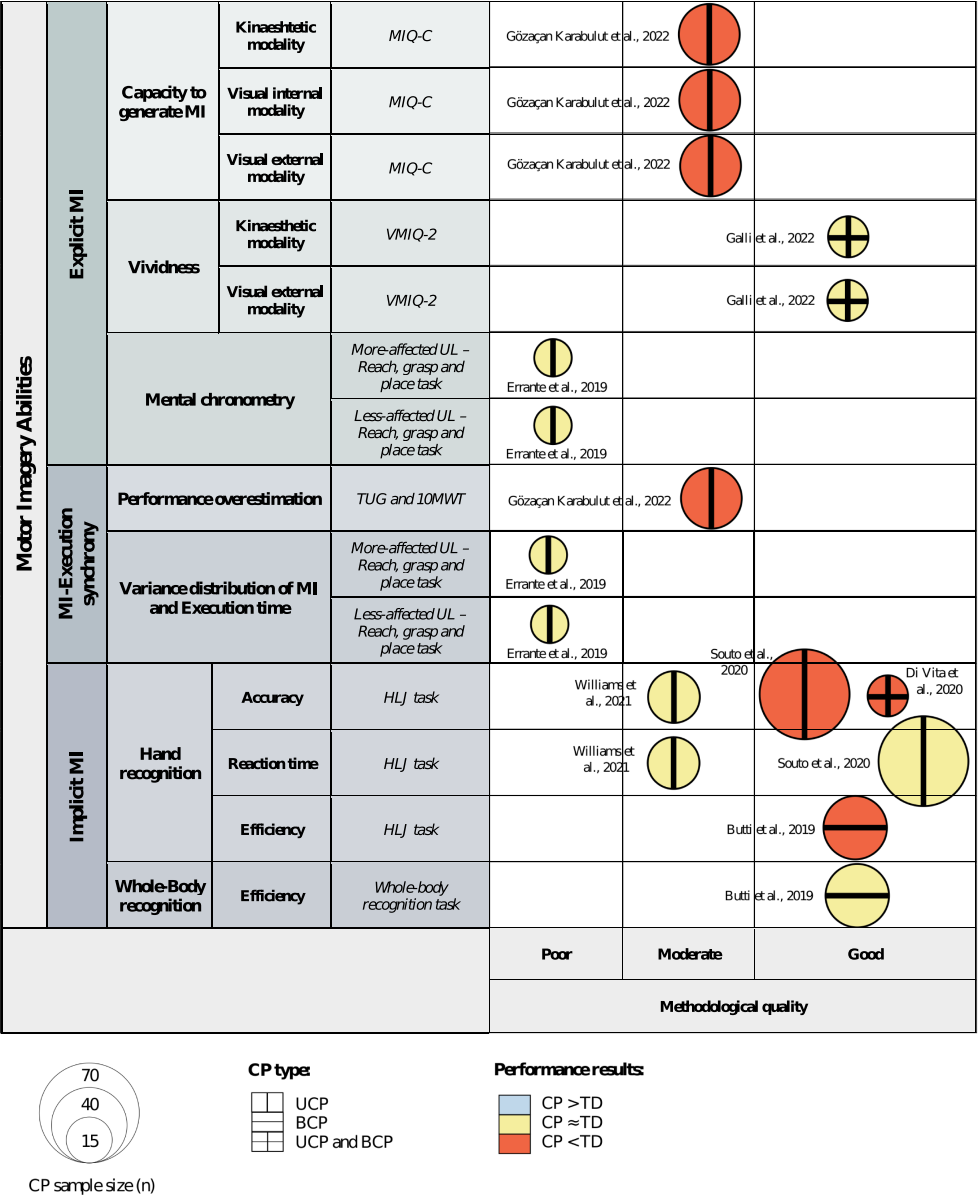
Qualitative evidence map of available evidence. Abbreviations: 10MWT, 10 meter walk test; BCP, bilateral cerebral palsy; CP, cerebral palsy; HLJ, hand laterality judgment; MI, motor imagery; MIQ-C, movement imagery questionnaire for children; TD, typically developed; TUG, timed-up-and-go test; UCP, unilateral cerebral palsy; UL, upper limb; VMIQ-2, vividness of movement imagery questionnaire 2nd version.

The qualitative evidence map synthesized the available information regarding methodological quality, outcome measures and measurement tools, sample size, CP population, and comparison results. See Figure 2.

### 3.6 Quantitative evidence mapping

**Figure 3:**
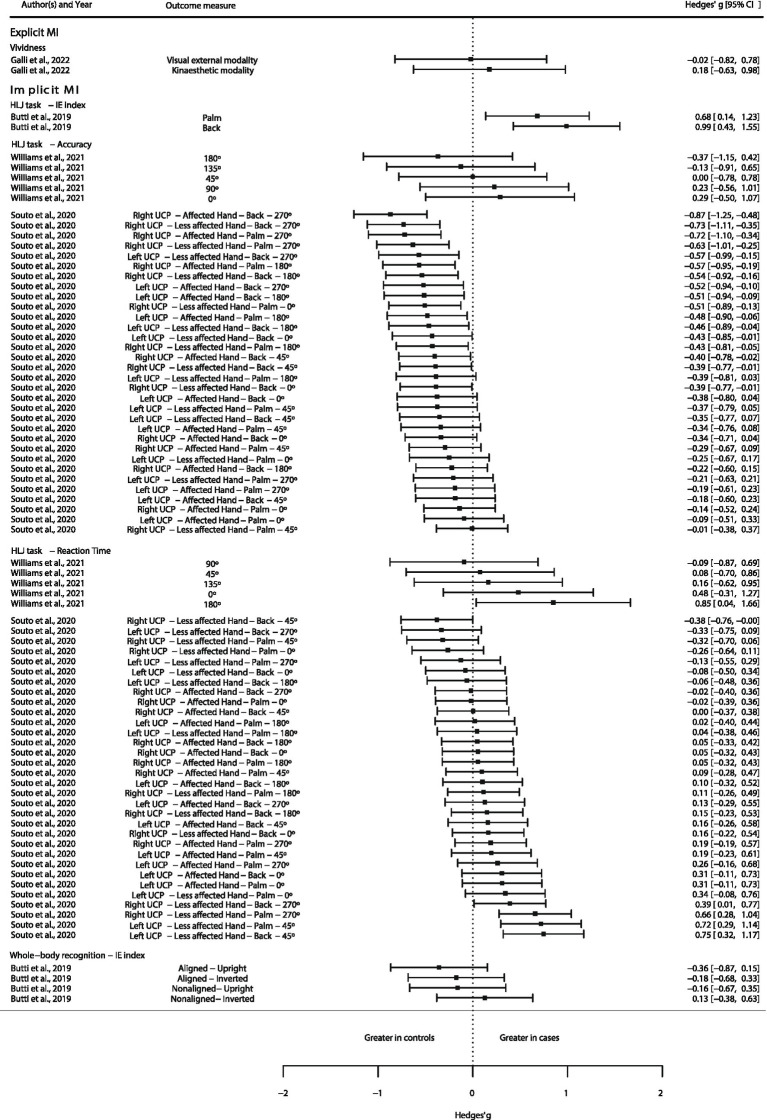
Quantitative evidence map of available evidence. Abbreviations: BCP, bilateral cerebral palsy; CP, cerebral palsy; HLJ, hand laterality judgment; IE, inverse efficiency; MI, motor imagery; UCP, unilateral cerebral palsy.

Authors were only able to extract quantitative data from four (41, 43–45) out of the seven studies that explored MI abilities between patients with CP and TD subjects. Data from three studies (41, 43, 44) were transformed from a standard error of the mean (SE) into SD, employing the following formula: $\left[SD\approx \sqrt{n}\times SE\right]$ proposed in the *Cochrane Handbook for Systematic Reviews of Interventions* section 6.5.2.2 (47). See Figure 3.

## 4 Discussion

This study aimed to gather and synthesize the evidence of MI abilities in children and adolescents with CP compared to TD subjects.

The evidence obtained from seven studies, considered for review in this study, poses significant limitations for drawing clear conclusions regarding explicit MI abilities in CP patients. Three different domains of explicit MI were assessed, such as the capacity to generate MI (29), vividness (45), and mental chronometry (44). There is moderate evidence to show that patients with CP and TD controls display similar vividness during MI. This finding stands out as the most reliable, whereas evidence concerning the capacity to generate MI and mental chronometry features between CP and TD controls is obviously limited.

Similarly, the current evidence concerning MI–execution synchrony is also limited (29, 42), clouding the direction of results between patients with CP and TD controls.

Conversely, implicit MI yields more straightforward interpretation of the results. A clear trend is observed for hand recognition accuracy (assessed with the HLJ task), with patients with CP displaying a lower accuracy than TD subjects. This observation is supported by moderate evidence (41, 46). There is also moderate evidence to show that patients with CP and TD controls exhibit similar reaction time values in the HLJ task (41). Only moderate evidence is available demonstrating a lower efficiency (the ratio between reaction time and accuracy) in the HLJ task (44). These findings align with the earlier studies, suggesting that reduced efficiency may stem from lower accuracy (41, 46), although similar reaction time is maintained (41) between patients with CP and TD controls.

Implicit MI ability for whole-body recognition appears to be similar between cases and controls, supported by moderate evidence (44).

Different interpretations and hypotheses may arise from the results obtained for the implicit MI ability for hand and whole-body recognition tasks. First, somatosensory body representations may be more impaired in children and adolescents with CP than TD subjects (48). Therefore, a more focused hand recognition task could point out detectable differences between the two groups, while a whole-body approach may not do so, suggesting a body-specific representation difference. Additionally, the lack of experience when employing upper limbs may limit the representation of these body regions, constraining the ability of recognition. In fact, sensorimotor cortex overactivations have been identified in children with CP compared to TD subjects when performing bimanual tasks (49), suggesting an association between the constraints during a bimanual task and the sensorimotor activity in order to perform the task.

The HLJ task has proven to be effective in assessing implicit MI, particularly in the context of brain damage. However, recent research indicates that the mental rotation involved in tasks like HLJ might not sufficiently conclude MI capacity in CP patients, which necessitate more explicit and targeted approaches (50, 51).

### 4.1 Neural insights and self-reported MI measures

As indicated by recent research, CP not only leads to deficits in movement execution but also to causes difficulties in motor planning (9) and MI.

MI involves the internal simulation of a movement without physical execution, activating sensorimotor circuits similar to those used during actual movements. Key areas such as the supplementary motor area, dorsal and ventral premotor cortices, and the inferior parietal lobule play a critical role in this process (52–54). Research suggests MI may be a valuable tool for motor function recovery in children with CP, though the ability of these to implement MI strategies might be compromised (55).

Understanding the impact of early brain damage and cerebral development on MI capacity is important beyond therapeutic implications. Studies suggest that damage to specific brain areas can disrupt children’s ability to effectively perform MI, restricting both the planning and execution of movements (56, 57). Previous findings indicate that children with right-sided congenital CP exhibit greater difficulties in MI tasks compared to their counterparts with left-sided congenital CP (58). Furthermore, it has been observed that individuals with right-sided congenital CP demonstrate deficits in anticipatory movement planning (59). These two deficits should be taken into account when devising motor imagery-based interventions for children with congenital CP.

To enhance our understanding of the capacity for MI, it is important to look into prior findings pertaining to cerebral behaviour inferred from neuroimaging studies for MI tasks. In this context, a previous study observed that patients with right-side early brain damage exhibited activation in the bilateral frontoparietal network, encompassing the majority of nodes associated with MI in healthy individuals. Conversely, patients with early left-side brain lesions demonstrated diminished cerebral activation during these tasks. Furthermore, there was only a minimal influence as regards the side of the imagined hand movement. This attenuated activation in patients with right UCP underscores the predominance of the left hemisphere in MI tasks (60).

In contrast to these findings, another study analysed brain activations during explicit MI in children with UCP and TD subjects (42). The results demonstrated that some children with UCP retained brain activations in cortical and subcortical areas during kinaesthetic MI tasks similar to those observed in TD children. Notably, a comparable parieto-frontal activation was observed in the right contralesional hemisphere during the imagination of reaching and grasping actions with the non-preferred hand. Furthermore, a correlation was noted between MI scores and the inferior parietal lobule and the dorsal sector of the premotor cortex were activated in both UCP and TD children, suggesting role for parietal activation in the online control of action execution. The study also highlighted the involvement of subcortical regions such as the putamen and cerebellum in explicit MI for complex grasping actions, indicating the engagement of a crucial cortico-basal-thalamic-cortical circuit in motor planning and learning. Interestingly, some children with UCP exhibited increased activations not only in the contralesional hemisphere but also in the ipsilesional one, particularly in those with primarily subcortical damage (42). The specificity of the employed MI modality, which involves imagining the action from a first-person perspective, might account for the discrepancy of these results with previous findings (42). These findings provide a neural foundation for integrating MI tasks into rehabilitation strategies for patients with UCP.

Adopting explicit MI strategies, such as mental chronometry, could provide a more accurate assessment of MI capacity and reveal specific deficiencies related to brain damage. Moreover, understanding how the activation of brain regions during MI tasks correlates with motor performance can offer valuable insights for designing targeted therapeutic interventions. As our understanding of the interaction between MI, cerebral development, and CP is deepened, improved rehabilitation strategies can be devised to optimize recovery and functionality in children affected by CP. The assessment of MI capacities in this child population could enhance our approach to prescribing MI-based therapies. For instance, gaining a comprehensive understanding of their capacity to generate MI and vividness across the three distinct MI modalities (kinaesthetic, visual internal, and visual external), or across MI perspectives (external or internal with no modality specified), would enable us to recommend the most effective approach. For example, prescribing MI through the modality or perspective in which the most patients with CP demonstrate greater capacities would be prioritized. Furthermore, comprehending their perceived difficulty and MI–execution synchrony across tasks of varying complexity could help us establish a progression order based on their performance. This involves starting with tasks perceived as less difficult, and where their MI–execution synchrony is closer, and gradually progressing to more complex tasks with a wider disparity between MI and its overt execution.

### 4.2 Limitations

All the studies included in this review had limitations with statistical procedures, mainly with sample size calculations, as no sample size calculations were performed (41–46, 61) or was not described in depth (29). This limitation poses difficulties for hypothesis testing and detecting the real amount of magnitude of difference between groups becomes difficult.

Additionally, the studies that analysed the ability to generate MI (29) and the vividness of MI (44) have methodological limitations due to the tools employed, MIQ-C an VMIQ-2, as they have only been validated in healthy children (62) and young athletes (24, 25), respectively. No validity processes have been conducted for these tools in children and adolescents with CP. Therefore, the certainty of the results may cannot be guaranteed. The findings of this systematic review highlight the need for further research on exploring the MI as a therapeutic tool for children with CP.

Therefore, the limitations mentioned above pose challenges for establishing a clear direction of the effect with regard to explicit MI and MI–execution synchrony domains. Nevertheless, a consistent direction could be ascertained for implicit MI abilities, especially hand and whole-body recognition, because of the availability of moderate evidence. Patients with CP present a lower capacity for hand recognition in the HLJ task, evident from a lower accuracy rate, but they maintain a similar reaction time as TD subjects. This leads to a reduced efficiency in hand recognition. This constraint could be specific for certain impaired body regions, such as the arms, as whole-body recognition features did not differ between the two groups.

## 5 Conclusion

Current research on MI abilities in children and adolescents with CP is scarce. Evidence is available for explicit MI domains like the capacity to generate MI, vividness, and mental chronometry, as well as MI–execution synchrony domains, and implicit MI domains such as hand and whole-body recognition. Notably, studies have significant limitations in sample size calculations, impacting the certainty of their results. However, a clear conclusion could be derived from implicit MI results, with moderate evidence suggesting that patients with CP present a reduced ability in hand recognition (HLJ tasks), but a similar capacity for whole-body recognition compared to TD controls. Previous research has questioned the validity of HLJ tasks in evaluating implicit MI. This has led to the proposal of alternative approaches like explicit MI or MI–execution synchrony tasks for assessing MI ability. The present review observed severe limitations for stating a clear direction with regard to explicit MI and MI-Execution synchrony domains. First, the absence of validated tools for assessing the capacity to generate MI and vividness (explicit MI domains) restricts the scope of their findings. Second, mental chronometry (explicit MI domain), performance overestimation, and MI–execution chronometry distribution (MI–execution synchrony domains) offer only limited evidence, posing difficulties in establishing a clear direction for their results. Future research should include improved research methodologies, including proper sample size calculations, and employ validated and reliable measurement procedures. A better understanding concerning MI abilities in patients with CP would lead to the development of tailored MI therapeutic interventions for them based on their strengths and the challenges they encounter.

## Data availability statement

The original contributions presented in the study are included in the article/Supplementary material, further inquiries can be directed to the corresponding author.

## Author contributions

JF-M: Data curation, Formal analysis, Methodology, Visualization, Writing – original draft, Writing – review & editing. AC-M: Data curation, Formal analysis, Methodology, Visualization, Writing – original draft. RL: Conceptualization, Formal analysis, Funding acquisition, Methodology, Project administration, Resources, Supervision, Writing – original draft, Writing – review & editing. SL-L: Funding acquisition, Project administration, Writing – review & editing, Conceptualization.

## Footnotes

^1^ https://apps.automeris.io/wpd/
