# Supplementary material for: Motor imagery ability in children and adolescents with cerebral palsy: a systematic review and evidence map
Source: Front Neurol. 2024 Feb 6;15:1325548. doi: 10.3389/fneur.2024.1325548 (PMC10876901; doi:10.3389/fneur.2024.1325548)
Supplement: Supplementary file 1 [file Table_1.DOCX]

**Supplementary Material.** Search and selection process: search engines, databases, search equations and results.

| **Search Engine** | **Databases** | **Searches (nº)** | **PICOS components** | | **Equation** | **Date** | **Registries (n)** |
| --- | --- | --- | --- | --- | --- | --- | --- |
|  |  |  | **Population** | **Outcome** |  |  |  |
| PubMed | MEDLINE | Nº1 | Cerebral palsy | MI | (“motor imagery”[Title/Abstract] OR “laterality”[Title/Abstract]) AND (“cerebral palsy”[Title/Abstract] OR “Cerebral Palsy”[Mesh]) | 7^th^ July, 2022 | 85 |
|  |  | Nº2 | Cerebral palsy | MI | (("cerebral palsy"[Title/Abstract] OR "Cerebral Palsy"[Mesh] OR ((“brain damage” [Title/Abstract] OR “brain injury”[Title/Abstract]) AND (“child*”[Title/Abstract] OR “adolescent*”[Title/Abstract] OR child[Filter] OR adolescent[Filter]))) AND (“motor imagery”[Title/Abstract] OR “laterality”[Title/Abstract] OR “movement imagery”[Title/Abstract] OR “mental imagery”[Title/Abstract] OR “visual imagery”[Title/Abstract] OR “kinesthetic imagery”[Title/Abstract] OR “kinaesthetic imagery”[Title/Abstract] OR “visuospatial imagery”[Title/Abstract] OR “guided imagery”[Title/Abstract] OR “skill imagery”[Title/Abstract] OR “mental practice”[Title/Abstract] OR “mental rehearsal”[Title/Abstract] OR “mental training”[Title/Abstract] OR “mental task”[Title/Abstract] OR “motor imagination”[Title/Abstract] OR “movement imagination”[Title/Abstract] OR “imagined movement”[Title/Abstract] OR “action imagination”[Title/Abstract] OR “imagined action”[Title/Abstract] OR “imagined task”[Title/Abstract] OR “mental chronometry”[Title/Abstract] OR “imagery duration”[Title/Abstract] OR “handedness”[Title/Abstract] OR “footedness”[Title/Abstract] OR “mental representation”[Title/Abstract] OR “motor representation”[Title/Abstract] OR “body schema”[Title/Abstract] OR “body representation”[Title/Abstract] OR “laterality judgement”[Title/Abstract] OR “laterality task”[Title/Abstract] OR “left right judgement”[Title/Abstract] OR “body recognition”[Title/Abstract])) NOT (("cerebral palsy"[Title/Abstract] OR "Cerebral Palsy"[Mesh]) AND ("motor imagery"[Title/Abstract] OR "laterality"[Title/Abstract]) AND 1600/01/01:2022/07/07[crdt]) | 13^th^ December, 2023 | 143 |
| EBSCO | Academic Search Premier; Education Source; ERIC; Library, Information Science & Technology Abstracts; MEDLINE Complete; OpenDissertations; PSICODOC; Sociology Source Ultimate; Teacher Reference Center; The Serials Directory | Nº1 | Cerebral palsy | MI | (AB motor imagery OR AB laterality) AND AB cerebral palsy | 7^th^ July, 2022 | 85 |
| Web of Science | Web of Science Core Collection; Current Contents Connect; Derwent Innovations Index; KCI-Korean Journal Database; MEDLINE; ProQuest Dissertations & Theses Citation Index; SciELO Citation Index | Nº1 | Cerebral palsy | MI | AB=(“cerebral palsy”) AND AB=(“motor imagery”) | 7^th^ July, 2022 | 35 |
|  |  | Nº2 | Cerebral palsy | MI | AB=(“cerebral palsy”) AND AB=(“laterality”) | 7^th^ July, 2022 | 52 |
| PEDro | - | Nº1 | Cerebral palsy | MI | “motor imagery” AND “cerebral palsy” | 7^th^ July, 2022 | 1 |
|  |  | Nº2 | Cerebral palsy | MI | “laterality” AND “cerebral palsy” | 7^th^ July, 2022 | 1 |
| Google Scholar | - | Nº1 | Cerebral palsy | MI | allintitle: (“motor imagery” OR “laterality”) AND “cerebral palsy”  *Filter: Including citations.* | 7^th^ July, 2022 | 16 |
